# Development and field trial of a *Tams1*-targeted isothermal DNA amplification (*Tams1*-lamp) assay for detection of *Theileria annulata *in cattle

**DOI:** 10.1186/1756-3305-7-S1-P5

**Published:** 2014-04-01

**Authors:** J Gomes, A Amaro, G Santos-Gomes, I Pereira da Fonseca, J Inácio

**Affiliations:** 1 Instituto Nacional de Investigação Agrária e Veterinária, I.P. Lisbon, Portugal; 2 Instituto de Higiene e Medicina Tropical, Lisbon, Portugal; 3 Faculdade de Medicina Veterinária, Universidade Técnica de Lisboa, Portugal

## 

Mediterranean Theileriosis is frequently diagnosed in cattle in southern Portugal. This tick-borne hemoprotozoan disease, caused by the apicomplexan parasite *Theileria annulata*, poses important health problems in cattle in addition to the economic losses caused by decrease of productivity in chronic infected animals. Integrated control strategies for this disease will then benefit to the detection of cattle with chronic infection that usually acts as parasite reservoirs constituting a risk to susceptible animals. The highly sensitive and specific molecular methods for *T. annulata *detection are suitable for the identification of such animals often with very low parasitaemia. The isothermal amplification methods proved particularly useful for diagnostic laboratories with fewer resources since it does not require the use of expensive and sophisticated equipment such as thermal cyclers. In the present study we develop an isothermal amplification technique targeting *Tams1 *gene (*Tams1*-LAMP) for detection of *T. annulata *in cattle blood samples and compared this technique with real time PCR for assessing its applicability for diagnosis. One hundred blood samples from 16 farms were collected and analyzed. Genomic DNA extracted from blood was used as template in real time PCR and LAMP reactions for *Tams1 *gene amplification. A DNA sample extracted from *T. annulata *cell culture and one from an animal with 0.03% parasitaemia were used as positive controls. DNA samples from other closely related parasites were used as negative controls. The LAMP technique detection limit has been determined to correspond to a parasitaemia of approximately 0.008% and a specificity of 100%. Real time PCR positive samples were detected in 14 farms with a total of 67% infected animals with very low parasitaemia that usually is characteristics of chronic infections. *Tams1*-LAMP identified 66% infected animals from the 14 farms. Cohen's test was used to evaluate the agreement between methods and the k value found (0.98) is indicative of an excellent agreement for this group of samples. These results permitted to conclude that *Tams1*-LAMP is a useful molecular technique to be applicable for detecting *Theileria annulata *in cattle with chronic infections.

